# A Principal Component Analysis of Metabolome and Cognitive Decline Among Japanese Older Adults: Cross-sectional Analysis Using Tohoku Medical Megabank Cohort Study Data

**DOI:** 10.2188/jea.JE20240099

**Published:** 2025-01-05

**Authors:** Sakura Kiuchi, Kumi Nakaya, Upul Cooray, Kenji Takeuchi, Ikuko N. Motoike, Naoki Nakaya, Yasuyuki Taki, Seizo Koshiba, Shunji Mugikura, Ken Osaka, Atsushi Hozawa

**Affiliations:** 1 Frontier Research Institute for Interdisciplinary Sciences, Tohoku University, Sendai, Japan; 2 Department of International and Community Oral Health, Tohoku University Graduate School of Dentistry, Sendai, Miyagi, Japan; 3 Tohoku Medical Megabank Organization, Tohoku University, Sendai, Japan; 4 Division of Epidemiology, School of Public Health, Graduate School of Medicine, Tohoku University, Sendai, Japan; 5 National Dental Research Institute Singapore, National Dental Centre Singapore, Singapore; 6 Division of Statistics and Data Science, Liaison Center for Innovative Dentistry, Tohoku University Graduate School of Dentistry, Sendai, Japan; 7 Systems Bioinformatics, Graduate School of Information Sciences, Tohoku University, Sendai, Japan; 8 Division of Health Behavioral Epidemiology, Tohoku University Graduate School of Medicine, Sendai, Japan; 9 Department of Aging Research and Geriatric Medicine, Institute of Development, Aging and Cancer, Tohoku University, Sendai, Japan; 10 The Advanced Research Center for Innovations in Next-Generation Medicine, Tohoku University, Sendai, Japan; 11 Department of Diagnostic Radiology, Graduate School of Medicine, Tohoku University, Sendai, Japan

**Keywords:** cognitive impairment, metabolomics, geriatrics

## Abstract

**Background:**

Dementia is the leading cause of disability and imposes a significant burden on society. Previous studies have suggested an association between metabolites and cognitive decline. Although the metabolite composition differs between Western and Asian populations, studies targeting Asian populations remain scarce.

**Methods:**

This cross-sectional study used data from a cohort survey of community-dwelling older adults aged ≥60 years living in Miyagi, Japan, conducted by Tohoku Medical Megabank Organization between 2013 and 2016. Forty-three metabolite variables quantified using nuclear magnetic resonance spectroscopy were used as explanatory variables. Dependent variable was the presence of cognitive decline (≤23 points), assessed by the Mini-Mental State Examination. Principal component (PC) analysis was performed to reduce the dimensionality of metabolite variables, followed by logistic regression analysis to calculate odds ratios (ORs) and 95% confidence intervals (CIs) for cognitive decline.

**Results:**

A total of 2,940 participants were included (men: 49.0%, mean age: 67.6 years). Among them, 1.9% showed cognitive decline. The first 12 PC components (PC1–PC12) accounted for 71.7% of the total variance. Multivariate analysis showed that PC1, which mainly represented essential amino acids, was associated with lower odds of cognitive decline (OR 0.89; 95% CI, 0.80–0.98). PC2, which mainly included ketone bodies, was associated with cognitive decline (OR 1.29; 95% CI, 1.11–1.51). PC3, which included amino acids, was associated with lower odds of cognitive decline (OR 0.81; 95% CI, 0.66–0.99).

**Conclusion:**

Amino acids are protectively associated with cognitive decline, whereas ketone metabolites are associated with higher odds of cognitive decline.

## INTRODUCTION

Dementia is the leading cause of disability and a significant burden for aging societies.^1^ In 2018, over 50 million individuals were estimated to be affected with dementia worldwide, and the numbers were projected to increase to 152 million by 2050.^2^ In addition, dementia and deterioration of cognitive function are a substantial burden for caregivers^3^^,^^4^ and healthcare systems.^4^^,^^5^ Therefore, understanding the underlying mechanisms of cognitive decline is an urgent requirement to identify solutions to reduce the burden of dementia.

Previous studies have suggested an association between metabolites and cognitive decline; metabolites can be predictors of cognitive decline.^6^^–^^13^ A systematic review and previous studies have shown that the concentration of several metabolites, such as lipids, branched-chain amino acids (BCAA; ie, valine, leucine, and isoleucine), steroids, and glucose levels, were associated with the onset of dementia progression.^6^^–^^9^ Another study reported that adding the metabolome data to the primary variables, such as sex and age, improved the predictability of mild cognitive impairment.^10^ For diagnosing Alzheimer’s disease, invasive procedures, such as cerebrospinal fluid and imaging biomarkers, are frequently used.^14^ Therefore, predicting cognitive decline from a relatively minimally invasive blood draw using the metabolome, especially at an early stage for detection and intervention, remains important.

When assessing the association between metabolites and cognitive decline, there is a lack of evidence that considers the metabolites as a pattern, rather than an individual metabolite. Moreover, the majority of the current evidence is based on cohort databases from European countries, such as the United Kingdom (UK)^7^^,^^9^ and North/Latin America,^10^^,^^12^ with little evidence from Asian populations.^13^^,^^15^ Because of their different diets and body structures, older adults in Asian and Western countries might have different metabolite compositions. Therefore, we aimed to investigate the association of the patterns of various metabolites, including essential amino acids, non-essential amino acids, glycolytic metabolites, and ketone bodies, with cognitive decline, using biobank data from Japanese community-dwelling older adults.

## METHODS

This cross-sectional study used baseline data from the Tohoku Medical Megabank Community-Based Cohort Study (TMM CommCohort Study), a sub-cohort of the TMM Project conducted in Miyagi and Iwate Prefectures in Japan from 2013 to 2016.^16^^–^^18^ The TMM project aims to contribute to the recovery of disaster damage from the Great East Japan Earthquake (March 11, 2011) and to build a next-generation healthcare system. The TMM CommCohort Study was conducted in collaboration with lifestyle health checkups, on-site examinations, and community support center examinations, targeting those aged ≥20 years. The TMM CommCohort Study conducted a questionnaire survey on demographic characteristics and lifestyles, examined physical activity, and obtained blood samples. All blood samples were stored at 4°C at the study site. The majority of the samples were transported within 8 hours and frozen at −80°C in accordance with the protocol.^19^ They were then cryopreserved until metabolomic analysis. The blood sampling period was from May 2013 to June 2016. The detailed procedures for the examination and blood sampling have been previously described.^18^ Metabolite measurements have been ongoing since 2015.^20^ In this study, we used the samples of metabolites measured up to January 2023.

Among the participants of the TMM CommCohort Study, we targeted community-dwelling older adults aged 60–89 years living in Miyagi Prefecture who were assessed for cognitive function. A cognitive function test was conducted from July 2014 to October 2019. Participants from the primary survey of the TMM CommCohort Study were recruited for these cognitive tests, which were administered to those who consented to participate. This cognitive function test was conducted as part of the TMM Brain Magnetic Resonance Imaging Study, and the details of the recruiting process have been described elsewhere.^21^ A total of 3,611 participants were included in the cognitive function test. Among them, 3,134 were eligible for our study participants. After excluding 194 participants with missing variables, such as age and metabolites, a total of 2,940 participants were included in the analytical sample.

### Dependent variable

Cognitive decline was assessed using the Mini-Mental Scale Examination (MMSE) as the dependent variable.^22^ The MMSE questionnaire consists of 20 items and is widely used for screening of dementia. Lower MMSE scores indicate worse cognitive function. We dichotomized this variable with cut-off scores of 23 and 24, based on a previous study.^23^

### Explanatory variables

Forty-three metabolites, including essential amino acids, non-essential amino acids, ketone bodies, glycolytic metabolites, and other metabolites obtained from blood samples, were used as dependent variables. Nuclear magnetic resonance (NMR) spectroscopy was used to quantify the metabolites in the blood samples.^24^ The plasma metabolites were analyzed using a non-target type method. The details of the NMR metabolome analysis have been previously described.^18^^,^^20^ eTable 1 shows a list of the metabolites used in the analysis. More information on the metabolites of this cohort is provided elsewhere (jMorp; https://jmorp.megabank.tohoku.ac.jp/).

### Covariates

Following previous studies and domain knowledge,^7^^,^^12^ we selected the covariates obtained from the questionnaire for analysis. Sex (men, women), age (60–64, 65–69, and ≥70 years), educational attainment (low [≤9], middle [10–12], and high [≥13 years]), body mass index (BMI; low [<18.5 kg/m^2^], normal [18.5–25.0 kg/m^2^], and obese [≥25.0 kg/m^2^]), presences of diabetes (yes or no), presence of hypertension (yes or no), and walking time (<30, 30–59, 60–179, and ≥180 min) as a proxy for physical activity, were included.

### Statistical analysis

For 43 metabolite variables, we performed the principal component analysis (PCA) to reduce the dimensionality and avoid multicollinearity.^25^^,^^26^ PCA was performed using centered and scaled metabolome data.^27^ After obtaining the PCA components, we performed a logistic regression analysis with PCA components and covariates to estimate odds ratios (ORs) and 95% confidence intervals (CIs) for cognitive decline.

We included 12 components obtained using PCA in the models, as Jolliffe and Cadima suggested that a cumulative variance of at least >70% should be retained.^26^ Among the 12 components, we especially focused on PC1–PC3. Model 1 included sex and age as the PCA components. In model 2, we included all covariates (sex, age, educational attainment, BMI, diabetes, hypertension, and walking time). We calculated the Variable Inflation Factor to check the multicollinearity of the variables. We applied random forest imputation to impute for missing values using “*missForest*” R package.^28^ Random forest imputation is commonly used for the metabolome data, particularly in cases where the types of missingness are unknown.^29^

We conducted several sensitivity analyses to check the robustness of the results. First, we conducted a different MMSE cut-off. We set the cut-off of the MMSE variable at 24/25 points as a more conservative cut-off compared with the main analysis to detect early cognitive decline. Second, we conducted the analysis treating the MMSE as a continuous variable with a linear regression model. Finally, we performed a sex-stratified analysis and an analysis stratified by age (70 years).

Statistical analyses were performed using R software (ver. 4.2.1; R Foundation for Statistical Computing, Vienna, Austria). We used the “*prcomp*” base R function for the PCA and the “*factoextra*” package for the visualizations. The Tohoku Medical Megabank Organization (ToMMo) supercomputer system provided the computational resources. We followed the Strengthening the Reporting of Observational Studies in Epidemiology (STROBE) guidelines for reporting.

### Ethical approval

The TMM CommCohort Study was approved by the Ethical Committee of ToMMo (first approval no. 2012-4-617 and latest approval no. 2023-4-134). Informed consent was obtained from all participants.

## RESULTS

Table 1 presents the descriptive characteristics of the study participants. A total of 2,940 participants were included in the analysis (men: 49.0%). The mean age of the participants was 67.6 (standard deviation, 4.2) years. The percentage of patients with cognitive decline was 1.9%. eTable 2 shows the descriptive characteristics after imputation and a similar distribution to that before imputation.

**Table 1.** Descriptive characteristics of the study sample (*n* = 2,940)

		Cognitive decline	
		No	Yes
Total		*n* = 2,885	*n* = 55
Sex	Men	1,413 (49.0)	29 (52.7)
	Women	1,472 (51.0)	26 (47.3)
Age, years	60–64	740 (25.6)	7 (12.7)
	65–69	1,278 (44.3)	18 (32.7)
	≥70	867 (30.1)	30 (54.5)
Educational attainment	Low	227 (7.9)	17 (30.9)
	Middle	1,586 (55.0)	30 (54.5)
	High	1,039 (36.0)	7 (12.7)
	Missing	33 (1.1)	1 (1.8)
Body mass index	Low	120 (4.2)	2 (3.6)
	Normal	2,076 (72.0)	39 (70.9)
	Obese	678 (23.5)	14 (25.5)
	Missing	11 (0.4)	0 (0.0)
Diabetes	No	1,700 (58.9)	29 (52.7)
	Yes	557 (19.3)	7 (12.7)
	Missing	628 (21.8)	19 (34.5)
Hypertension	No	1,288 (44.6)	19 (34.5)
	Yes	1,234 (42.8)	28 (50.9)
	Missing	363 (12.6)	8 (14.5)
Walking time, mins	<30	358 (12.4)	7 (12.7)
	30–59	923 (32.0)	16 (29.1)
	60–179	1,108 (38.4)	18 (32.7)
	≥180	427 (14.8)	9 (16.4)
	Missing	69 (2.4)	5 (9.1)

Note: Cognitive decline was measured by Mini Mental State Examination (MMSE).

The cut off of MMSE is 23/24 point.

**Figure 1.**
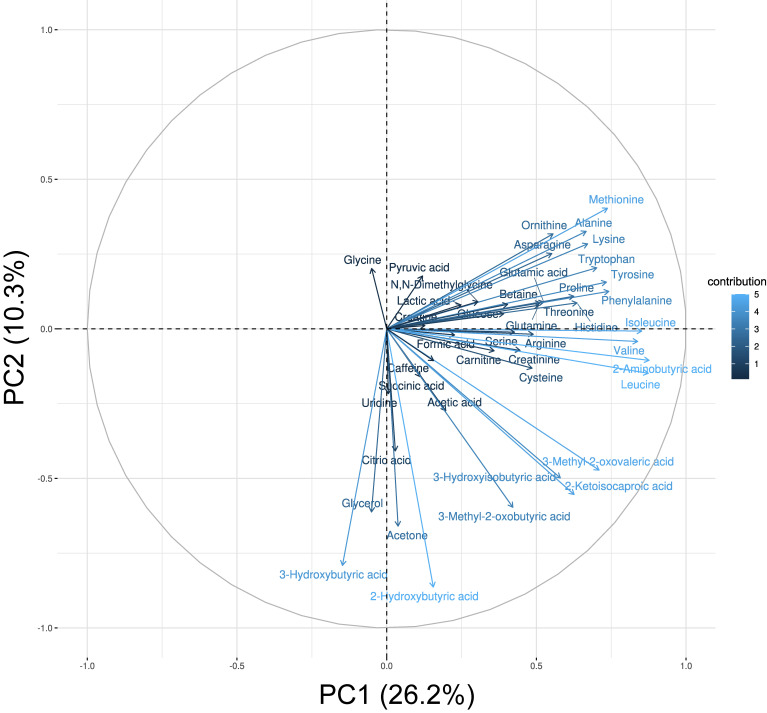
Biplot of PC1 and PC2 for principal component analysis. PC, principal component.

**Figure 2.**
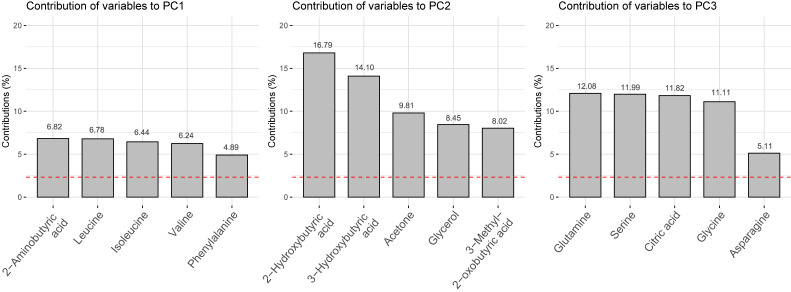
The top five loadings contributing to PC1, PC2 and PC3. PC, principal component.

**Figure 3.**
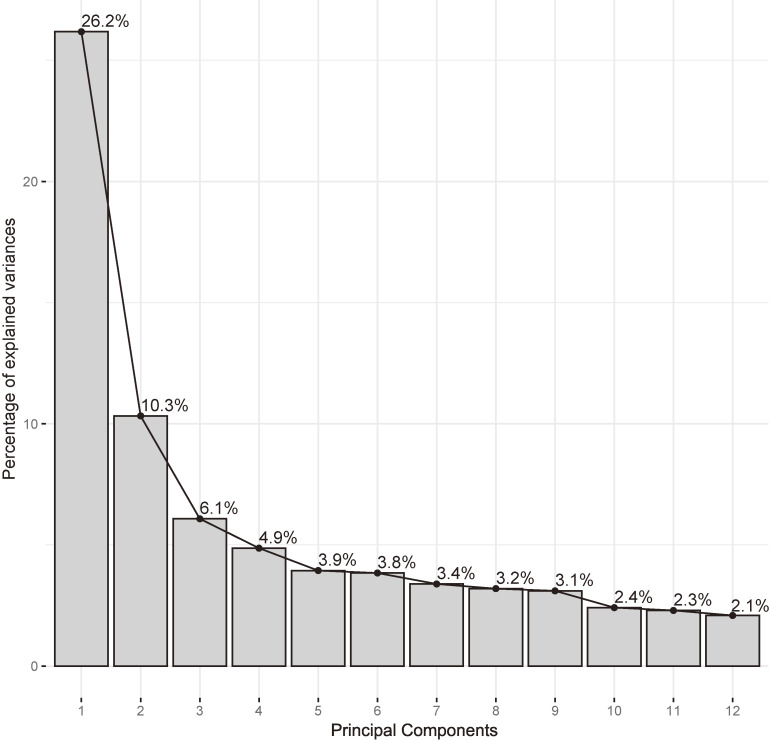
Scree plot for the percentage of explained variances

Figure 1 and Figure 2 present the PCA results. Figure 1 shows a biplot of PC1 and PC2. Figure 2 shows the top five loadings for the contributions from PC1 to PC3. PC1 (26.2%) included 2-aminobutyric acid, leucine, isoleucine, valine, and phenylalanine (essential amino acid pattern). PC2 (10.3%) included ketone bodies, glycerol, 2-hydroxybutyric acid, 3-hydroxybutyric acid, acetone, glycerol, and 3-methyl-2-oxobutyric acid (ketone-rich pattern). PC3 (6.1%) included glutamine, serine, citric acid, glycine, and asparagine (amino acid pattern). The biplots of PC1 and PC3 and PC2 and PC3 are shown in eFigure 1. The results for PC4–PC12 are shown in eFigure 2. The details of the loadings identified by PCA are shown in Table 2 (PC1–PC3) and eTable 3 (PC4–PC12). The cumulative variance of PC1–PC12 for the total variability was 71.7% (Figure 3).

**Table 2.** Factor loadings for major metabolites components identified by principal components analysis

		PC1	PC2	PC3
Essential amino acids	Methionine	**0.220**	**0.191**	0.042
	Tryptophan	**0.209**	0.097	0.020
	Isoleucine	**0.254**	−0.004	0.080
	Phenylalanine	**0.221**	0.059	−0.040
	Histidine	**0.189**	0.041	**−0.176**
	Leucine	**0.260**	−0.071	0.116
	Lysine	**0.200**	0.135	−0.083
	Threonine	**0.153**	0.041	**−0.194**
	Valine	**0.250**	−0.020	**0.152**
Non-essential amino acids	2-Aminobutyric acid	**0.261**	−0.050	0.110
	3-Methyl-2-oxovaleric acid	**0.211**	**−0.224**	0.141
	2-Ketoisocaproic acid	**0.187**	**−0.263**	**0.156**
	Creatine	0.038	0.005	−0.145
	Cysteine	0.145	−0.062	0.009
	Glutamic acid	**0.156**	0.042	**0.209**
	Creatinine	0.133	−0.034	−0.047
	Asparagine	**0.164**	0.120	**−0.226**
	Arginine	0.146	−0.009	−0.076
	Ornithine	**0.166**	**0.151**	−0.079
	Tyrosine	**0.219**	0.074	0.014
	Serine	0.127	−0.005	**−0.346**
	Proline	**0.186**	0.052	0.042
	Glycine	−0.015	0.096	**−0.333**
	Alanine	**0.199**	**0.155**	0.047
	Glutamine	**0.151**	0.037	**−0.348**
	3-Methyl-2-oxobutyric acid	0.126	**−0.283**	0.149
	Betaine	0.120	0.039	−0.117
	N,N-Dimethylglycine	0.091	0.043	−0.033
Ketone bodies	Acetone	0.011	**−0.313**	−0.092
	3-Hydroxyisobutyric acid	**0.173**	**−0.237**	0.102
	2-Hydroxybutyric acid	0.046	**−0.410**	0.022
	Acetic acid	0.059	−0.130	−0.109
	Carnitine	0.107	−0.035	−0.108
	3-Hydroxybutyric acid	−0.044	**−0.375**	−0.145
Glycolytic metabolites	Succinic acid	0.033	−0.076	**−0.203**
	Pyruvic acid	0.036	0.084	0.147
	Glycerol	−0.015	**−0.291**	−0.113
	Lactic acid	0.074	0.038	0.038
	Glucose	0.117	0.024	0.036
	Citric acid	0.009	**−0.194**	**−0.344**
	Formic acid	0.068	−0.010	−0.025
	Caffeine	0.047	−0.051	−0.011
	Uridine	0.002	−0.103	**−0.195**

PC, principal component.

Note: The bold fonts mean the absolute values <−0.15 or >0.15.

Table 3 shows the results of the logistic regression analysis. After adjustments of covariates, PC1 (essential amino acid pattern) was significantly associated with lower odds of cognitive decline (OR 0.89; 95% CI, 0.80–0.98). PC2 (ketone-rich pattern) was associated with higher odds of cognitive decline (OR 1.29; 95% CI, 1.11–1.51). PC3 (amino acid pattern) was associated with lower odds of cognitive decline (OR 0.81; 95% CI, 0.66–0.99). PC8, which included creatine, creatinine, carnitine, formic acid, and succinic acid, was associated with higher odds of cognitive decline (OR 1.43; 95% CI, 1.10–1.87), and the other PCs were not associated with cognitive decline. eTable 4 shows the results of the Variable Inflation Factor of the analysis.

**Table 3.** Association between principal components from metabolites and cognitive decline among the participants (*n* = 2,940)

	Model 1				Model 2			

	OR	95% CI		*P*-value	OR	95% CI		*P*-value
PC1	**0.91**	**0.82**	**1.00**	**0.047**	**0.89**	**0.80**	**0.98**	**0.021**
PC2	**1.31**	**1.13**	**1.54**	**0.001**	**1.29**	**1.11**	**1.51**	**0.001**
PC3	**0.85**	**0.71**	**1.03**	**0.102**	**0.81**	**0.66**	**0.99**	**0.041**
PC4	1.13	0.94	1.36	0.177	1.11	0.92	1.33	0.293
PC5	1.04	0.85	1.27	0.702	1.04	0.85	1.28	0.697
PC6	0.95	0.76	1.19	0.643	0.94	0.75	1.19	0.624
PC7	1.03	0.83	1.27	0.804	1.01	0.80	1.26	0.942
PC8	**1.42**	**1.09**	**1.83**	**0.008**	**1.43**	**1.10**	**1.87**	**0.008**
PC9	1.18	0.92	1.52	0.185	1.20	0.93	1.56	0.165
PC10	1.30	0.97	1.75	0.081	1.30	0.96	1.76	0.092
PC11	0.92	0.70	1.22	0.581	0.94	0.71	1.23	0.638
PC12	1.01	0.76	1.35	0.928	1.00	0.75	1.33	0.981

(Intercept)	**0.43**	**0.19**	**0.95**	**<0.001**	**0.37**	**0.16**	**0.84**	**<0.001**

AUC	0.73				0.80			

AUC, area under the curve; CI, confidence interval, OR: odds ratio; PC, principal component.

Note: Model 1 included sex and age as covariates.

Model 2: Educational attainment, body mass index, diabetes, hypertension and walking time were adjusted in addition to Model 1.

The bold fonts mean the statistical significance.

eTable 5 and eTable 6 show the results of the sensitivity analysis using a more conservative MMSE cut-off (24/25 points). The directions of the results from the sensitivity analysis were similar to the main findings, although only PC2 was significantly associated with cognitive decline (OR 1.13; 95% CI, 1.03–1.25). eTable 7 shows the results of the linear regression model with the dependent variable as a continuous variable. The trends of the results were similar to the main findings, and PC1, PC2, and PC8 showed statistical significance. eTable 8 shows the results of the sex-stratified analysis. PC1, PC2 and PC8 showed statistical significance in women; however, no PCs show statistical significance in men. eTable 9 shows the results of stratified analysis divided by age. PC1, PC2 and PC8 showed statistical significance in those aged ≥70 years, but only PC8 shows statistical significance in those aged <70 years.

## DISCUSSION

This study examined the association between metabolite patterns and cognitive decline in Japanese community-dwelling older adults. Our findings suggest that the patterns of predominance of essential and other amino acids possibly protect against cognitive decline. A ketone-rich pattern was associated with higher odds of cognitive decline. These findings suggest that amino acid and ketone metabolites are important indicators of cognitive decline in older Japanese populations. Moreover, these findings are important for understanding the mechanisms underlying the association between metabolites and cognitive decline.

Our findings are consistent with previous studies that reported essential amino acids were protectively associated with cognitive decline.^6^^–^^8^^,^^15^ A study using eight prospective cohort studies and another study using the UK biobank showed that essential amino acids were associated with a lower risk of Alzheimer’s disease.^6^^,^^7^ A systematic review also reported this association.^8^ In addition, a previous Japanese study reported that lower levels of essential amino acids were associated with the development of Alzheimer’s disease among participants with mild cognitive impairment.^15^ Regarding the association between ketone metabolites and cognitive decline, our findings showed a similar tendency to a previous study that analyzed UK biobank data.^7^ As most previous studies targeting large populations have been conducted in Western countries,^6^^–^^8^ this is the first study to find that higher amino acids and lower ketone metabolites are associated with cognitive decline among Asian older adults. Our findings indicate that metabolite patterns are reliable indicators of cognitive decline among the older population in Japan, which is one of the fastest-aging Asian countries.

Our study contributes to the existing literature by reporting findings from Asian countries with low obesity rates and diets that differ from those in Western countries. The prevalence of obesity is lower in Japan than in the countries where most of the previous studies were conducted.^30^ The Japanese diet among older adults is characterized by a high intake of soybean products, fish, vegetables, seaweed, and green tea, which are rich sources of antioxidants and omega-3 fatty acids.^31^ Diet-induced metabolism could be different from the dietary habits of Western countries, which include high levels of processed foods and saturated fats. However, our results support the previous studies, even after accounting for obesity, diabetes, and hypertension.

The possible mechanisms underlying the association between amino acid metabolites, including essential amino acids and cognitive decline, are as follows: amino acids act as precursors of neurotransmitters in the brain and are responsible for energy production.^32^^,^^33^ From a review study that investigated protein intake and cognitive decline, the authors suggested that amino acid intake leads to improved sleep, quality exercise, controlled stress, and decreased depression and anxiety, which can protect against cognitive decline.^34^ Particularly, BCAA is involved in the synthesis and breakdown of skeletal muscles, which also affects the progression of sarcopenia and decreases physical activity.^15^^,^^35^ Lower physical activity is a known risk factor for cognitive decline.^4^ Therefore, individuals with lower amino acid levels are at risk of cognitive decline.

The possible mechanisms underlying the association between ketone metabolites and cognitive decline are as follows: ketone bodies are produced by the metabolism of fatty acids and ketogenic amino acids when the glucose supply is restricted, such as during starvation.^7^^,^^36^ A higher supply of ketone bodies is an indicator of poor nutritional status, and insufficient glucose supply to the brain may cause cognitive decline. However, some studies have reported that increased plasma ketone levels might inhibit the progression of Alzheimer’s disease, although most findings come from animal studies.^37^ The detailed mechanisms underlying the association between ketone bodies and cognitive decline remain unknown and further investigation is needed.

Our results showed that PC8 was associated with higher odds of cognitive decline. PC8 primarily included creatine and creatinine levels. Creatine is a compound that promotes the provision of energy to the muscle and brain tissue.^38^ Creatinine is a marker of renal function; higher levels of creatinine indicate renal dysfunction. Contrary to our results, creatine was suggested to be effective in improving cognitive function, and recent randomized controlled trials targeting young populations have reported that creatine supplementation is useful for cognitive performance.^39^ Conversely, the effect of creatine supplementation on cognitive decline and Alzheimer’s disease remains unknown.^40^ Whether creatine or creatinine can be a marker of cognitive function requires further investigation. However, PC8 accounted for only 3.2% of the total variance in the metabolites, which was not as high as that of PC1–PC3 (Figure 2). PC8 showed statistically significant, but the actual effect might be small.

Sensitivity analyses showed a variety of associations between metabolite components and cognitive decline. The sex-stratified analysis showed significant differences in PC1, PC2, and PC8 among women, but not among men. Age-stratified analysis showed a significant difference for PC8 for those aged <70 years, but PC1, PC2, and PC8 for those aged ≥70 years. The reduced sample size due to stratification may have contributed to the reduced power of detection; however, there is the possibility of a difference in the effectiveness between sex and age. This should be explored in future studies. Moreover, only ketone metabolites showed a significant difference when using a more conservative cut-off value than the main result. It indicates that ketone metabolites may be strong predictors of cognitive decline.

This study has two main important public health implications. First, our study indicates the importance of maintaining essential amino acid levels through a balanced diet. Essential amino acids can be obtained from meat, poultry, eggs, dairy products, fish, and other supplements. However, whether amino acid supplementation and protein intake are effective in protection against cognitive decline remains controversial.^34^ Future studies should explore the association between amino acid supplementation and cognitive decline. Second, our study suggests the possibility of predicting cognitive decline using blood metabolome data in Asian populations. The ability to detect cognitive decline through blood samples could lead to the development of less invasive assessment tools. Identifying metabolic markers that contribute to cognitive decline would also be useful for developing personalized interventions. Although we observed that lower amino acid levels and higher ketone body levels in the blood were associated with cognitive decline, further research is necessary to determine whether these markers can reliably predict or influence cognitive decline.

This study has several limitations. First, our study used cross-sectional data; therefore, we could not determine the temporality of the association based on our results. Essential amino acid intake may be reduced among those with cognitive decline because essential amino acids can only be obtained from food and cannot be synthesized in the body.^6^ Therefore, future studies with a longitudinal design are required to develop a predictive tool for cognitive decline. Second, we could not consider the possible confounders, such as genetic information and dietary intake. The possibility of genetic factors influencing cognitive function has been reported.^41^ Dietary intake may influence the essential amino acids; therefore, further research considering them is required. Third, the dates for collecting blood samples and the cognitive function test were different depending on the participants because participants were recruited to perform cognitive function tests after blood sampling (average: 1,175.3 days). Therefore, metabolome profiling may not directly reflect cognitive decline, particularly if the physical condition changes after blood samples are collected. Fourth, it is possible that our study population has better cognitive function than the general community-dwelling older population in Japan. The prevalence of cognitive decline was 1.9% in our study, but the previous systematic review reported the global prevalence of cognitive impairment among community-dwelling adults aged ≥50 years was 15.6%.^42^ A previous Japanese study with a similar setting reported 10.9% as the prevalence of cognitive decline.^43^ As the mean age of this study’s participants was older than that of our study, we could not compare directly. However, this difference could be due to the recruiting process or possibly other factors, such as area, study design, and measurements. Therefore, our findings may not be generalizable, especially for non-Japanese populations. Fifth, we started measuring metabolites 2 years after the first blood samples were taken. Therefore, cryopreservation could affect the composition of the metabolites.^44^ However, we made a protocol,^19^ transported most of the samples within 8 hours, and stored the blood at −80°C, which is considered as best practice.^45^

The strengths of this study are as follows: first, our study included a relatively large number of participants. Second, studies on metabolites and cognitive decline, particularly those targeting older Asian adults, are lacking. These findings are important for understanding the mechanisms underlying the association between metabolites and cognitive decline in Asian populations. Third, we used metabolites measured using NMR spectroscopy by trained staff. Metabolites measured using NMR spectroscopy are known to be quantitative and involve simpler procedures for sample preparation than mass spectrometry.^46^

### Conclusion

Amino acid metabolites are protectively associated with cognitive decline, whereas ketone metabolites are associated with higher odds of cognitive decline in the older Japanese population. Metabolome monitoring can be useful for predicting and preventing future cognitive decline.

## SUPPLEMENTARY MATERIAL

eFigure 1. Biplots of PC1 and PC3 and PC2 and PC3 for principal component analysis. PC, principal component

eFigure 2. The top five loadings contributing to PC4–PC12. PC, principal component

eTable 1. List of the metabolites included in the analysis

eTable 2. Descriptive characteristics of the study sample after imputation (n = 2,940)

eTable 3. Factor loadings for metabolites components (PC4–PC12) identified by principal components analysis

eTable 4. Variance Inflation Factor for the analysis with the cut-off of MMSE (23/24 points) (n = 2,940)

eTable 5. Descriptive characteristics of the study sample with different MMSE cut-off values (24/25 points) (n = 2,940)

eTable 6. Association between principal components from metabolites and cognitive decline among the participants with different cut-off of MMSE values (24/25 points) (n = 2,940)

eTable 7. Association between principal components from metabolites and cognitive decline as continuous variable among the participants (n = 2,940)

eTable 8. Association between principal components from metabolites and cognitive decline among the participants stratified by sex (n = 2,940)

eTable 9. Association between principal components from metabolites and cognitive decline among the participants stratified by age (n = 2,940)
