# Supplementary material for: A Principal Component Analysis of Metabolome and Cognitive Decline Among Japanese Older Adults: Cross-sectional Analysis Using Tohoku Medical Megabank Cohort Study Data
Source: J Epidemiol. 2025 Jan 5;35(1):39–46. doi: 10.2188/jea.JE20240099 (PMC11637816; doi:10.2188/jea.JE20240099)
Supplement: Supplementary file 1 [file je-35-039-s001.pdf]

eTable 1. List of the metabolites included in the analysis

Amino acids	Essential amino acids	Methionine
		Tryptophan
		Isoleucine
		Phenylalanine
		Histidine
		Leucine
		Lysine
		Threonine
		Valine
	Non-essential amino acids	2-Aminobutyric acid
		3-Methyl-2-oxovaleric acid
		2-Ketoisocaproic acid
		Creatine
		Cysteine
		Glutamic acid
		Creatinine
		Asparagine
		Arginine
		Ornithine
		Tyrosine
		Serine
		Proline
		Glycine
		Alanine
		Glutamine
		3-Methyl-2-oxobutyric acid
		Betaine
		N,N-Dimethylglycine
Ketone bodies		Acetone
		3-Hydroxyisobutyric acid

	2-Hydroxybutyric acid
	Acetic acid
	Carnitine
	3-Hydroxybutyric acid
Glycolytic metabolites	Succinic acid
	Pyruvic acid
	Glycerol
	Lactic acid
	Glucose
	Citric acid
Other metabolites	Formic acid
	Caffeine
	Uridine

eTable 2. Descriptive characteristics of the study sample after imputation (n=2,940)

		Cognitive decline	
		No	Yes
Total		n=2,885	n=55
Sex	Men	1,413 (49.0)	29 (52.7)
	Women	1,472 (51.0)	26 (47.3)
Age, years	60–64	740 (25.6)	7 (12.7)
	65–69	1,278 (44.3)	18 (32.7)
	≥70	867 (30.1)	30 (54.5)
Educational attainment	Low	227 (7.9)	17 (30.9)
	Middle	1,599 (55.4)	31 (56.4)
	High	1,059 (36.7)	7 (12.7)
BMI	Low	120 (4.2)	2 (3.6)
	Normal	2,087 (72.3)	39 (70.9)
	Obese	678 (23.5)	14 (25.5)
Diabetes	No	2,012 (69.7)	37 (67.3)
	Yes	873 (30.3)	18 (32.7)
Hypertension	No	1,290 (44.7)	19 (24.5)
	Yes	1,595 (55.3)	36 (65.5)
Walking time, mins	<30	358 (12.4)	7 (12.7)
	30–59	923 (32.0)	16 (29.1)
	60–179	1,176 (40.8)	23 (41.8)
	≥180	428 (14.8)	9 (16.4)

BMI, body mass index.

eTable 3. Factor loadings for metabolites components (PC4-PC12) identified by principal components analysis

		PC4	PC5	PC6	PC7	PC8	PC9	PC10	PC11	PC12
Essential amino acids	Methionine	**0.220**	**0.191**	0.042	**-0.171**	0.004	0.061	-0.008	-0.003	0.014
	Tryptophan	**0.209**	0.097	0.020	0.016	-0.117	-0.041	0.110	0.094	-0.059
	Isoleucine	**0.254**	-0.004	0.080	-0.015	0.136	0.124	-0.064	-0.039	0.042
	Phenylalanine	**0.221**	0.059	-0.040	0.026	0.042	-0.018	**0.156**	0.063	0.023
	Histidine	**0.189**	0.041	**-0.176**	-0.084	-0.119	-0.063	0.105	-0.135	-0.142
	Leucine	**0.260**	-0.071	0.116	-0.064	0.084	0.051	-0.067	-0.018	0.047
	Lysine	**0.200**	0.135	-0.083	**-0.161**	0.063	**-0.215**	0.039	-0.126	0.135
	Threonine	**0.153**	0.041	**-0.194**	0.142	-0.142	0.077	**-0.256**	**0.326**	-0.160
	Valine	**0.250**	-0.020	**0.152**	-0.011	0.109	0.096	-0.036	-0.044	0.023
Non-essential amino acids	2-Aminobutyric acid	**0.261**	-0.050	0.110	-0.047	0.118	0.079	-0.039	0.011	0.000
	3-Methyl-2-oxovaleric acid	**0.211**	**-0.224**	0.141	-0.080	-0.100	0.125	0.006	0.049	-0.006
	2-Ketoisocaproic acid	**0.187**	**-0.263**	**0.156**	-0.148	**-0.178**	0.072	0.033	0.009	-0.009
	Creatine	0.038	0.005	-0.145	0.109	**0.485**	-0.147	0.102	**0.165**	**0.183**
	Cysteine	0.145	-0.062	0.009	0.106	-0.064	**-0.333**	0.028	-0.132	**0.294**
	Glutamic acid	**0.156**	0.042	**0.209**	**0.182**	**0.160**	**-0.176**	**-0.335**	-0.080	-0.126
	Creatinine	0.133	-0.034	-0.047	-0.038	**-0.423**	**-0.150**	-0.139	**-0.196**	**0.283**
	Asparagine	**0.164**	0.120	**-0.226**	**-0.176**	0.015	-0.051	-0.001	-0.015	-0.079
	Arginine	0.146	-0.009	-0.076	0.121	0.001	-0.127	**0.218**	**-0.421**	**0.324**
	Ornithine	**0.166**	**0.151**	-0.079	**-0.197**	**0.188**	-0.052	**-0.179**	0.004	-0.008
	Tyrosine	**0.219**	0.074	0.014	0.095	0.122	0.062	**0.163**	0.145	-0.045
	Serine	0.127	-0.005	**-0.346**	**0.192**	-0.071	**0.254**	-0.061	0.052	0.060
	Proline	**0.186**	0.052	0.042	**0.176**	0.002	0.079	**-0.260**	-0.092	-0.050
	Glycine	-0.015	0.096	**-0.333**	-0.001	0.083	**0.306**	**-0.273**	0.002	**0.319**
	Alanine	**0.199**	**0.155**	0.047	**0.161**	-0.120	**0.218**	0.052	-0.036	-0.042
	Glutamine	**0.151**	0.037	**-0.348**	**-0.164**	-0.036	0.064	-0.036	-0.120	**-0.245**
	3-Methyl-2-oxobutyric acid	0.126	**-0.283**	0.149	**-0.173**	**-0.156**	0.118	0.049	0.097	-0.103
	Betaine	0.120	0.039	-0.117	0.062	-0.136	**-0.270**	**0.183**	**0.233**	0.022
	N,N-Dimethylglycine	0.091	0.043	-0.033	**-0.154**	-0.022	**-0.234**	0.059	**0.568**	0.137
Ketone bodies	Acetone	0.011	**-0.313**	-0.092	0.028	0.044	**-0.152**	**-0.368**	-0.017	0.065

	3-Hydroxyisobutyric acid	**0.173**	**-0.237**	0.102	-0.026	0.128	0.017	**0.154**	0.087	0.094
	2-Hydroxybutyric acid	0.046	**-0.410**	0.022	0.007	0.113	-0.001	0.046	0.078	0.038
	Acetic acid	0.059	-0.130	-0.109	-0.052	0.038	**-0.227**	**-0.221**	**-0.156**	**-0.318**
	Carnitine	0.107	-0.035	-0.108	**0.282**	**-0.241**	**-0.270**	-0.119	**0.156**	-0.065
	3-Hydroxybutyric acid	-0.044	**-0.375**	-0.145	-0.066	0.121	-0.040	**-0.196**	-0.003	0.050
Glycolytic metabolites	Succinic acid	0.033	-0.076	**-0.203**	**-0.208**	**0.232**	-0.039	0.134	-0.123	0.042
	Pyruvic acid	0.036	0.084	0.147	**-0.183**	0.079	0.038	0.010	0.020	-0.058
	Glycerol	-0.015	**-0.291**	-0.113	**0.248**	0.017	0.063	**0.177**	-0.080	-0.086
	Lactic acid	0.074	0.038	0.038	**0.156**	**0.204**	0.029	-0.047	0.005	-0.049
	Glucose	0.117	0.024	0.036	**0.164**	-0.125	0.145	**0.171**	**-0.162**	-0.120
	Citric acid	0.009	**-0.194**	**-0.344**	-0.113	0.019	**0.231**	**0.186**	0.004	-0.056
	Formic acid	0.068	-0.010	-0.025	**0.347**	**0.233**	-0.143	0.147	-0.100	**-0.364**
	Caffeine	0.047	-0.051	-0.011	**0.377**	-0.027	**0.185**	0.043	0.137	**0.283**
	Uridine	0.002	-0.103	**-0.195**	-0.010	-0.099	**-0.167**	**0.182**	0.011	**-0.177**

PC, principal component.

Note: The bold fonts mean the absolute values <-0.15 or >0.15.

eTable 4. Variance Inflation Factor for the analysis with the cut off of MMSE (23/24 points) (n=2,940)

	Model 1	Model 2
PC1	1.25	1.30
PC2	1.05	1.06
PC3	1.13	1.19
PC4	1.04	1.05
PC5	1.00	1.02
PC6	1.08	1.09
PC7	1.02	1.03
PC8	1.15	1.15
PC9	1.08	1.10
PC10	1.06	1.07
PC11	1.02	1.03
PC12	1.02	1.04
Sex	1.49	1.52
Age	1.02	1.03
Educational attainment		1.01
BMI		1.04
Diabetes		1.12
Hypertension		1.12
Walking time		1.02

BMI, body mass index; MMSE, Mini Mental State Examination; PC, principal component.

Note: Model 1 included sex and age as covariates.

Model 2: Educational attainment, BMI, diabetes, hypertension and walking time in addition to Model 1.

eTable 5. Descriptive characteristics of the study sample with different cut-off of MMSE (24/25 points) (n=2,940)

		Cognitive decline	
		No	Yes
Total		n=2,819	n=121
Sex	Men	1,374 (48.7)	68 (56.2)
	Women	1,445 (51.3)	53 (43.8)
Age, years	60–64	731 (25.9)	16 (13.2)
	65–69	1,247 (44.2)	49 (40.5)
	≥70	841 (29.8)	56 (46.3)
Educational attainment	Low	215 (7.6)	29 (24.0)
	Middle	1,547 (54.9)	69 (57.0)
	High	1,025 (36.4)	21 (17.4)
	Missing	32 (1.1)	2 (1.7)
BMI	Low	120 (4.3)	2 (1.7)
	Normal	2,028 (71.9)	87 (71.9)
	Obese	660 (23.4)	32 (26.4)
	Missing	11 (0.4)	0 (0.0)
Diabetes	No	1,674 (59.4)	55 (45.5)
	Yes	539 (19.1)	25 (20.7)
	Missing	606 (21.5)	41 (33.9)
Hypertension	No	1,268 (45.0)	39 (32.2)
	Yes	1,201 (42.6)	61 (50.4)
	Missing	350 (12.4)	21 (17.4)
Walking time, mins	<30	339 (12.0)	26 (21.5)
	30–59	913 (32.4)	26 (21.5)
	60–179	1,086 (38.5)	40 (33.1)
	≥180	414 (14.7)	22 (18.2)
	Missing	67 (2.4)	7 (5.8)

BMI, body mass index; MMSE, Mini Mental State Examination.

eTable 6. Association between principal components from metabolites and cognitive decline among the participants with different cut-off of MMSE values (24/25 points) (n=2,940)

	Model 1				Model 2			
	OR	95% CI		P-value	OR	95% CI		P-value
PC1	0.98	0.91	1.04	0.448	0.96	0.89	1.02	0.194
PC2	**1.15**	**1.05**	**1.27**	**0.003**	**1.13**	**1.03**	**1.25**	**0.011**
PC3	0.96	0.84	1.09	0.497	0.92	0.81	1.06	0.240
PC4	1.04	0.92	1.18	0.539	1.01	0.89	1.14	0.915
PC5	0.95	0.83	1.09	0.447	0.95	0.83	1.09	0.468
PC6	0.94	0.81	1.09	0.400	0.94	0.80	1.09	0.414
PC7	1.12	0.97	1.30	0.113	1.10	0.94	1.27	0.231
PC8	**1.20**	**1.01**	**1.44**	**0.038**	1.20	1.00	1.43	0.054
PC9	1.03	0.87	1.21	0.753	1.04	0.88	1.23	0.669
PC10	1.13	0.94	1.37	0.201	1.12	0.92	1.36	0.248
PC11	0.93	0.77	1.12	0.441	0.94	0.78	1.13	0.519
PC12	0.94	0.78	1.14	0.546	0.95	0.79	1.15	0.632
(Intercept)	**0.03**	**0.01**	**0.05**	**<0.001**	**0.05**	**0.01**	**0.22**	**<0.001**
AUC	0.67				0.73			

AUC, area under the curve; CI, confidence interval; MMSE, Mini Mental State Examination; OR, odds ratio; PC, principal component.

Note: Model 1 included sex and age as covariates.

Model 2: Educational attainment, body mass index, diabetes, hypertension and walking time in addition to Model 1.

The bold fonts mean the statistical significance.

eTable 7. Association between principal components from metabolites and cognitive decline as continuous variable among the participants (n=2,940)

	Model 1				Model 2			
	B coef.	95% CI		P-value	B coef.	95% CI		P-value
PC1	0.02	0.00	0.04	0.090	**0.03**	**0.01**	**0.05**	**0.007**
PC2	**-0.05**	**-0.08**	**-0.02**	**<0.001**	**-0.04**	**-0.07**	**-0.01**	**0.004**
PC3	0.02	-0.03	0.06	0.477	0.04	-0.01	0.08	0.104
PC4	-0.04	-0.08	0.01	0.105	-0.02	-0.07	0.02	0.271
PC5	0.03	-0.02	0.08	0.267	0.02	-0.03	0.06	0.477
PC6	0.03	-0.03	0.08	0.330	0.02	-0.03	0.07	0.518
PC7	-0.01	-0.06	0.04	0.706	0.00	-0.05	0.05	0.998
PC8	**-0.07**	**-0.13**	**-0.01**	**0.031**	**-0.06**	**-0.12**	**0.00**	**0.046**
PC9	-0.03	-0.08	0.03	0.329	-0.04	-0.09	0.02	0.161
PC10	-0.03	-0.09	0.04	0.400	-0.02	-0.08	0.04	0.525
PC11	-0.02	-0.08	0.05	0.591	-0.02	-0.09	0.04	0.447
PC12	0.01	-0.05	0.08	0.681	0.01	-0.05	0.08	0.672
(Intercept)	**28.27**	**28.10**	**28.43**	**<0.001**	**27.52**	**27.08**	**27.97**	**<0.001**

B coef: B coefficient; CI, confidence interval; PC, principal component.

Note: Model 1 included sex and age as covariates.

Model 2: Educational attainment, body mass index, diabetes, hypertension and walking time were adjusted in addition to Model 1.

The bold fonts mean the statistical significance.

eTable 8. Association between principal components from metabolites and cognitive decline among the participants stratified by sex (n=2,940)

	Men (n=1,442)								Women (n=1,498)							
	Model 1				Model 2				Model 1				Model 2			
	OR	95% CI		P-value	OR	95% CI		P-value	OR	95% CI		P-value	OR	95% CI		P-value
PC1	0.89	0.77	1.02	0.117	0.89	0.76	1.03	0.134	0.89	0.77	1.03	0.123	**0.85**	**0.73**	**0.99**	**0.046**
PC2	**1.25**	**1.03**	**1.57**	**0.035**	1.16	0.95	1.46	0.158	**1.41**	**1.12**	**1.82**	**0.005**	**1.42**	**1.13**	**1.85**	**0.005**
PC3	0.80	0.61	1.05	0.102	0.80	0.60	1.05	0.109	0.85	0.64	1.14	0.284	0.75	0.54	1.03	0.082
PC4	1.04	0.80	1.33	0.791	1.00	0.76	1.30	0.983	1.19	0.90	1.59	0.230	1.16	0.86	1.58	0.323
PC5	0.98	0.75	1.29	0.883	0.98	0.73	1.30	0.864	1.09	0.81	1.49	0.587	1.10	0.81	1.52	0.562
PC6	0.81	0.59	1.09	0.170	0.81	0.59	1.10	0.181	1.14	0.81	1.63	0.454	1.14	0.79	1.64	0.480
PC7	1.10	0.82	1.47	0.521	1.05	0.77	1.41	0.763	0.99	0.70	1.39	0.974	1.00	0.68	1.43	0.982
PC8	1.24	0.87	1.77	0.229	1.24	0.84	1.81	0.272	**1.78**	**1.18**	**2.72**	**0.007**	**1.88**	**1.22**	**2.94**	**0.005**
PC9	1.33	0.95	1.89	0.104	1.45	1.01	2.11	0.051	1.05	0.71	1.56	0.798	1.03	0.69	1.54	0.881
PC10	1.29	0.87	1.92	0.211	1.36	0.90	2.08	0.146	1.28	0.82	2.05	0.289	1.28	0.81	2.07	0.291
PC11	1.00	0.69	1.42	0.988	1.00	0.70	1.40	0.988	0.83	0.51	1.31	0.432	0.86	0.52	1.39	0.544
PC12	1.06	0.72	1.60	0.759	1.04	0.70	1.56	0.861	0.99	0.61	1.58	0.958	0.97	0.60	1.55	0.903
(Intercept)	**0.01**	**0.00**	**0.04**	**<0.001**	0.10	0.00	1.22	0.109	**0.00**	**0.00**	**0.01**	**<0.001**	**0.00**	**0.00**	**0.04**	**<0.001**
AUC	0.72				0.84				0.78				0.81			

AUC, area under the curve; CI, confidence interval; OR, odds ratio; PC, principal component.

Note: Model 1 included sex and age as covariates.

Model 2: Educational attainment, body mass index, diabetes, hypertension and walking time were adjusted in addition to Model 1.

The bold fonts mean the statistical significance.

eTable 9. Association between principal components from metabolites and cognitive decline among the participants stratified by age (n=2,940)

	<70 years old (n=2,043)								≥70 years old (n=897)							
	Model 1				Model 2				Model 1				Model 2			
	OR	95% CI		P-value	OR	95% CI		P-value	OR	95% CI		P-value	OR	95% CI		P-value
PC1	0.92	0.78	1.06	0.257	0.92	0.77	1.07	0.287	**0.87**	**0.76**	**0.99**	**0.044**	**0.86**	**0.75**	**0.99**	**0.041**
PC2	1.18	0.96	1.50	0.135	1.16	0.94	1.48	0.189	**1.44**	**1.16**	**1.82**	**0.002**	**1.41**	**1.13**	**1.80**	**0.004**
PC3	0.88	0.66	1.16	0.364	0.85	0.63	1.15	0.297	0.82	0.63	1.07	0.145	0.80	0.60	1.06	0.119
PC4	1.23	0.93	1.62	0.144	1.19	0.91	1.59	0.216	1.05	0.80	1.36	0.705	1.05	0.80	1.37	0.701
PC5	0.92	0.69	1.23	0.556	0.96	0.70	1.31	0.803	1.15	0.87	1.54	0.321	1.11	0.83	1.50	0.478
PC6	0.96	0.68	1.35	0.836	1.03	0.71	1.48	0.875	0.94	0.69	1.27	0.681	0.87	0.63	1.19	0.374
PC7	1.09	0.79	1.50	0.594	1.10	0.78	1.54	0.585	1.00	0.73	1.36	0.978	0.98	0.70	1.36	0.907
PC8	1.48	0.97	2.25	0.067	**1.54**	**1.00**	**2.39**	**0.049**	**1.42**	**1.00**	**2.02**	**0.048**	**1.45**	**1.00**	**2.09**	**0.048**
PC9	1.05	0.73	1.51	0.813	1.08	0.74	1.58	0.707	1.27	0.90	1.83	0.181	1.27	0.88	1.87	0.212
PC10	1.36	0.89	2.11	0.157	1.36	0.88	2.14	0.180	1.30	0.87	1.97	0.203	1.31	0.87	2.01	0.207
PC11	0.76	0.49	1.14	0.199	0.77	0.49	1.18	0.239	1.13	0.76	1.64	0.536	1.10	0.76	1.60	0.607
PC12	0.78	0.50	1.21	0.266	0.74	0.47	1.16	0.194	1.25	0.83	1.90	0.297	1.25	0.83	1.90	0.299
(Intercept)	**0.01**	**0.00**	**0.03**	**<0.001**	0.00	0.00	2E+14	0.985	**0.04**	**0.02**	**0.07**	**<0.001**	**0.06**	**0.00**	**0.57**	**0.030**
AUC	0.73				0.82				0.74				0.79			

AUC, area under the curve; CI, confidence interval; OR, odds ratio; PC, principal component.

Note: Model 1 included sex and age as covariates.

Model 2: Educational attainment, body mass index, diabetes, hypertension and walking time were adjusted in addition to Model 1.

The bold fonts mean the statistical significance.

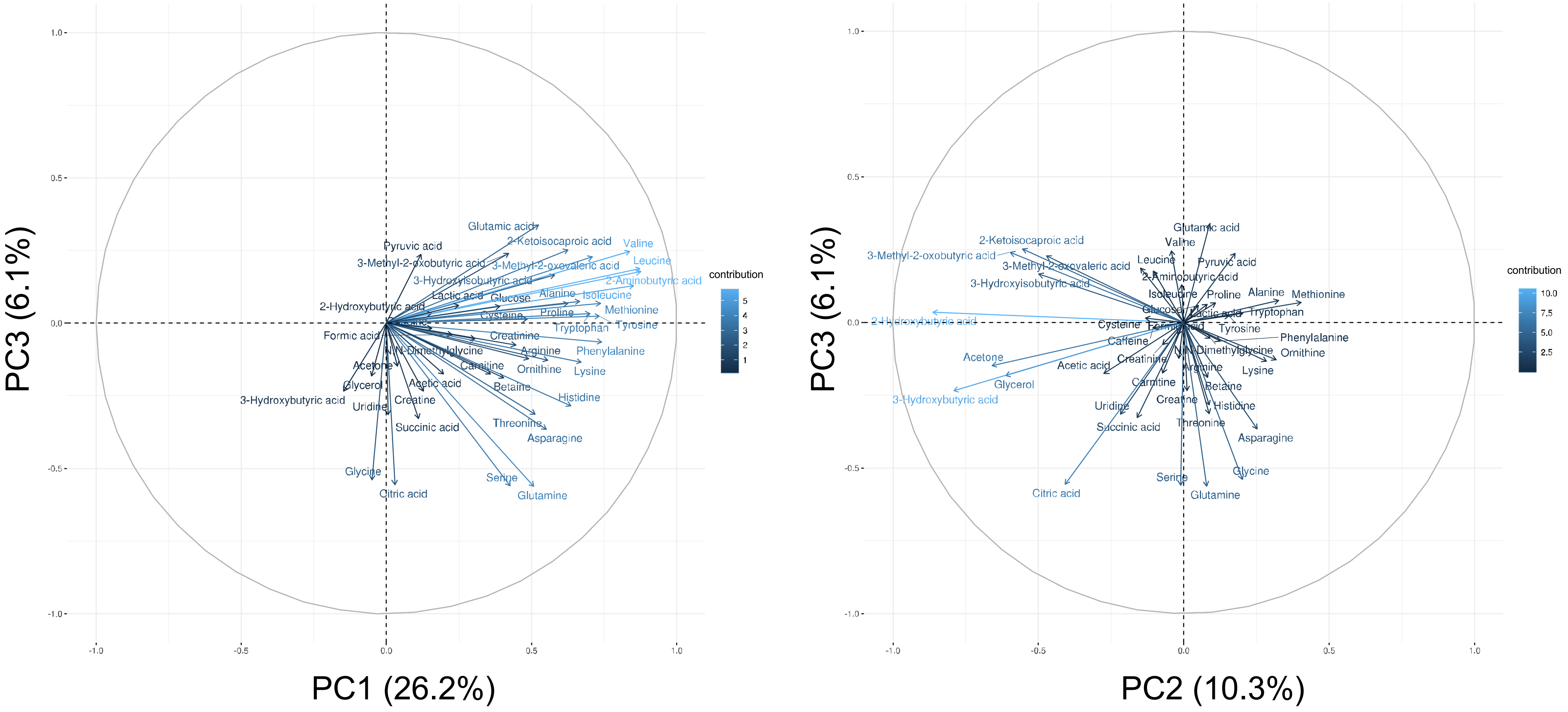

eFigure 1. Biplots of PC1 and PC3 and PC2 and PC3 for principal component analysis. PC, principal component

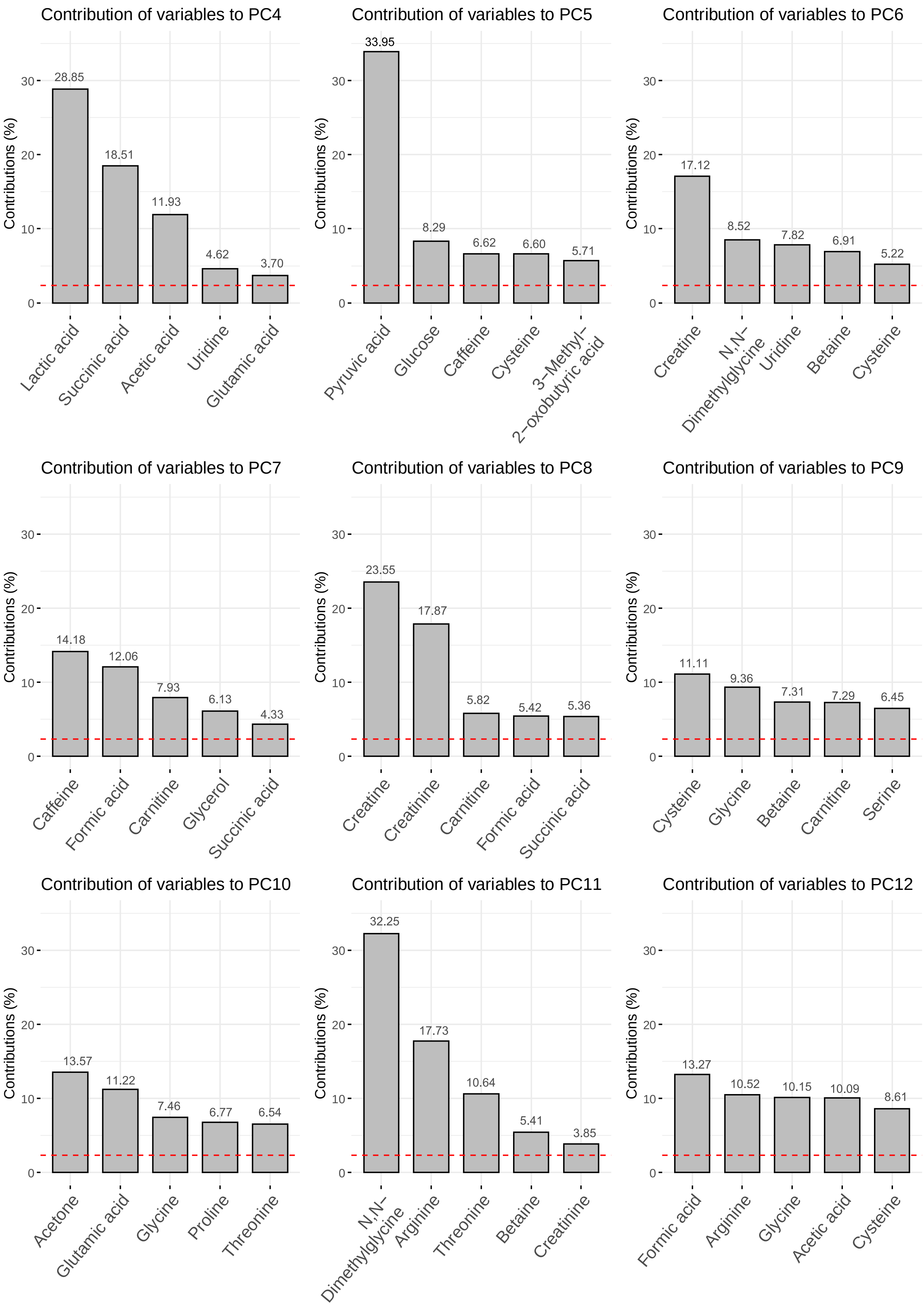

eFigure 2. The top five loadings contributing to PC4–PC12. PC, principal component
